# Stability and p*K*_a_ Modulation of Aminophenoxazinones and Their Disulfide Mimics by Host–Guest Interaction with Cucurbit[7]uril. Direct Applications in Agrochemical Wheat Models

**DOI:** 10.1021/acs.jafc.2c06373

**Published:** 2022-12-22

**Authors:** Francisco J. R. Mejías, Suhang He, Rosa M. Varela, José M. G. Molinillo, Andrea Barba-Bon, Werner M. Nau, Francisco A. Macías

**Affiliations:** † Department of Organic Chemistry, Institute of Biomolecules (INBIO), University of Cádiz, República Saharaui 7, Puerto Real11510, Spain; ‡ Department of Life Sciences and Chemistry, Jacobs University Bremen, Campus Ring 1, Bremen28759, Germany

**Keywords:** aminophenoxazinones, cucurbiturils, pK_a_ shift, disulfide, agrochemicals, host−guest interactions

## Abstract

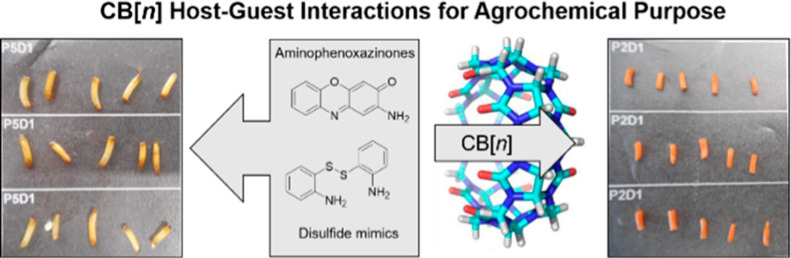

Aqueous solubility and stability often limit the application of aminophenoxazinones and their sulfur mimics as promising agrochemicals in a sustainable agriculture inspired by allelopathy. This paper presents a solution to the problem using host–guest complexation with cucurbiturils (**CB*n***). Computational studies show that **CB7** is the most suitably sized homologue due to its strong affinity for guest molecules and its high water solubility. Complex formation has been studied by direct titrations monitored using UV–vis spectroscopy, finding a preferential interaction with protonated aminophenoxazinone species with high binding affinities (**CB7·APOH**^**+**^, *K*_a_ = (1.85 ± 0.37) × 10^6^ M^–1^; **CB7·DiS-NH**_**3**_^**+**^, *K*_a_ = (3.91 ± 0.53) × 10^4^ M^–1^; and **DiS-(NH**_**3**_^**+**^**)**_**2**_, *K*_a_= (1.27 ± 0.42) × 10^5^ M^–1^). NMR characterization and stability analysis were also performed and revealed an interesting p*K*_a_ modulation and stabilization by cucurbiturils (2-amino-3*H*-phenoxazin-3-one (**APO**), p*K*_a_ = 2.94 ± 0.30, and **CB7·APO,** p*K*_a_ = 4.12 ± 0.15; 2,2′-disulfanediyldianiline (**DiS-NH**_**2**_), p*K*_a_ = 2.14 ± 0.09, and **CB7·DiS-NH**_**2**_, p*K*_a_ = 3.26 ± 0.09), thus favoring applications in different kinds of crop soils. Kinetic studies have demonstrated the stability of the **CB7·APO** complex at different pH media for more than 90 min. An in vitro bioassay with etiolated wheat coleoptiles showed that the bioactivity of **APO** and **DiS-NH**_**2**_ is enhanced upon complexation.

## 1 Introduction

Crop growth stimulation and protection are the main focal points identified by the European Union to secure future food production. Agriculture has become increasingly dependent on pesticides and persistent chemicals over the past few years, and the effect of this can currently be observed with soil pollution and resistance phenomena due to the continuous mode of action inherent to classical herbicides. Natural products have recently arisen as potential replacements, given their short half-lives and new modes of action, which allow a rebranding approach to weed control.^1^ Successful results have been observed in different weed species and genera with a broad variety of natural product families, for example, aminophenoxazinones. Application of 2-amino-3*H*-phenoxazin-3-one (**APO**) to *Arabidopsis thaliana* showed inhibition values of around 100%, whereas suberoylanilide hydroxamic acid, which was used as a positive control, gave values of only 75% at a high concentration of 200 μM.^2^

A common problem associated with the large-scale application of natural products is their poor water solubility and soil stability, which limit effective chemical transport and result in insufficient lifetimes to affect inhibition in the target weed. One promising strategy to solve this problem is the encapsulation of those agrochemicals in the water-soluble host via host–guest interactions. Cyclodextrins,^3^ organic nanoparticles,^4^ and nanotubes^5^ have been selected as host candidates and have afforded outstanding results in aqueous media when applied to phytotoxic or antibacterial compounds.

Cucurbit[*n*]uril (**CB*n***), a well-studied synthetic macrocyclic host family in supramolecular chemistry, has shown relevant applications in medicinal chemistry and drug delivery, for example, **CB7** has been used to complex with oxaliplatin, ferrocene derivatives, and curcumin to treat different kinds of tumors.^6^ In addition, **CB7** is the prototypical **CB*n*** homologue employed in the biological field due to its high water solubility in comparison to the other homologues of interest (*n* = 6 and 8). Indeed, the concentration of **CB7** in neat water can reach as high as 5 mM, while **CB6** and **CB8** only dissolve on a micromolar scale.

Despite their potential biological applications, comparatively few studies have been devoted to the exploitation of cucurbiturils in an agricultural context. For example, Saleh et al. applied **CB7** and **CB8** to generate a complex with fuberidazole in order to inhibit the growth of the fungus *Botrytis cinerea*.^8^ Similarly, Liu et al. applied the same methodology with **CB8** and carboxin to counteract the fungus *Rhizoctonia solani*,^9^ and Huang et al. encapsulated adefovir in **CB7** to enhance its activity against tobacco mosaic virus.^10^ Another direct application of cucurbiturils in agrochemistry is the encapsulation of classic herbicides, such as 2,4-D.^11^ In this work, we have studied the complexation of cucurbiturils and aminophenoxazinones for the first time in order to develop a potentially green application of natural products as new promising agrochemical formulations and to replace polluting herbicides. To achieve this goal, the physicochemical properties of natural compounds and mimics need to be modulated to optimize their efficacy. Media stability, water solubility, spectroscopic characterization, and biological evaluation of the complexes are analyzed and discussed in this study.

## 2 Experimental Section

### 2.1 General Chemicals

**CB7** was synthesized and purified in conc. HCl as described previously.^12^ The amount and content for **CB7** synthesis can be observed in Table S1. 2-Amino-3*H*-phenoxazin-3-one (**APO**) was synthesized following the method described by Macías et al.^13^ 2,2′-Disulfanediyldianiline (**DiS-NH**_**2**_) was synthesized following the method described by Oliveira et al.^14^

### 2.2 Measurements

UV–vis absorption measurements were performed using a Varian Cary 4000 spectrophotometer. All spectroscopic measurements were performed in 0.5 and 3.5 mL quartz cuvettes from Hellma Analytics (Müllheim, Germany) or cuvettes from Sigma-Aldrich (Steinheim, Germany). All solutions were prepared in fresh Millipore water, and the pH values were adjusted by adding small aliquots of HCl or NaOH solution. The acidity was read as the pH value using a WTW 330i pH meter equipped with a combined pH glass electrode (SenTix Mic).

^1^H NMR spectra were recorded in D_2_O at room temperature (r.t.) using a Bruker Avance NEO 400 MHz instrument equipped with a 5 mm QNP direct detection probe and *z*-gradients using the standard parameter set provided by Bruker. The chemical shifts are reported as δ values (ppm) relative to the residual peak of the solvent.

Spectral simulations and structural calculations were performed using Gaussian16 software and applying the B3LYP DFT method with the LanL2DZ basis set. The van der Waals volumes of the molecules were calculated using HyperChem version 8.0.10. The chemical structures of the compounds were optimized by MM2 energy minimization included in Chem3D before the volume calculation.

In the titration experiments, the concentration of the guest molecules was kept constant and that of **CB7** was increased gradually. The absorption spectra were recorded as a function of total **CB7** concentration at the appropriate wavelength. The binding constants (*K*_a_) were subsequently fitted with Origin software using the following user-defined formula which has been described previously^7^
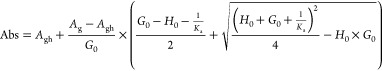
where Abs = absorbance of the testing solution at fixed λ, *G*_0_ = total concentration of the guest (analyte), *H*_0_ = total concentration of the host, *K*_a_ = binding constant, *A*_g_ = initial absorbance of the guest (analyte), and *A*_gh_ = absorbance of the complexed guest at concentration of *G*_0_.

The method to measure the binding constants is slightly different if we are measuring the p*K*_a_ shift or if we are measuring the complexation with the neutral molecule. In the case of neutral molecules, the guest molecule added in the quartz tray is previously dispersed in DMSO, but the concentration of this solvent never exceeds 0.5% of the total volume employed. In the case of the normal tray, the starting solution in the tray is prepared with 12.5 μL of DMSO + 2487.5 μL of H_2_O. When p*K*_a_ shift experiments are carried out, no DMSO is added. Solubility is enhanced, while pH is modified, and **CB*n*** host the molecule. Normal titration starts by adding 0.5 μL of one solution with the macrocycle and the same concentration of the guest molecule in the tray to avoid the dilution effect. After that, 1, 2, 4, 8, 16, 32, 64, 128, 256, and 512 μL are the usual volumes added to the tray, but the volume is decided according to the absorbance observed. In the case of a high binding constant, the volume increase between additions is smaller, to obtain a better representation of the titration curve.

### 2.3 In Vitro Etiolated Wheat Coleoptile Bioassay

All experiments were carried out following the procedure reported in the literature, with some modifications.^15^ Briefly, all free bioactive molecules and free macrocycles were predissolved in dimethyl sulfoxide (DMSO), 0.5% v/v, and subsequently diluted in an aqueous citric acid/dipotassium phosphate buffer containing 2% sucrose at pH 4.6, 5.6, or 6.6. On the other hand, complexes (**CB7·APOH**^**+**^, **CB7·DiS-NH**_**3**_^**+**^, and **CB7·DiS-(NH**_**3**_^**+**^**)**_**2**_) are solved only in buffer solution. The pH of the buffer in every bioassay was adjusted with sodium citrate/citric acid. For each pH, a series of testing solutions were prepared with different concentrations of the bioactive compound or guest analyte in the case of the complexes (10, 30, 100, 300, and 1000 μM). The concentrations of the resulting complexes were calculated based on the bioactive compound concentration. Osmotic stress was analyzed to validate the results. Logran was employed as the positive control in the bioassay.

## 3 Results and Discussion

**Figure 1:**
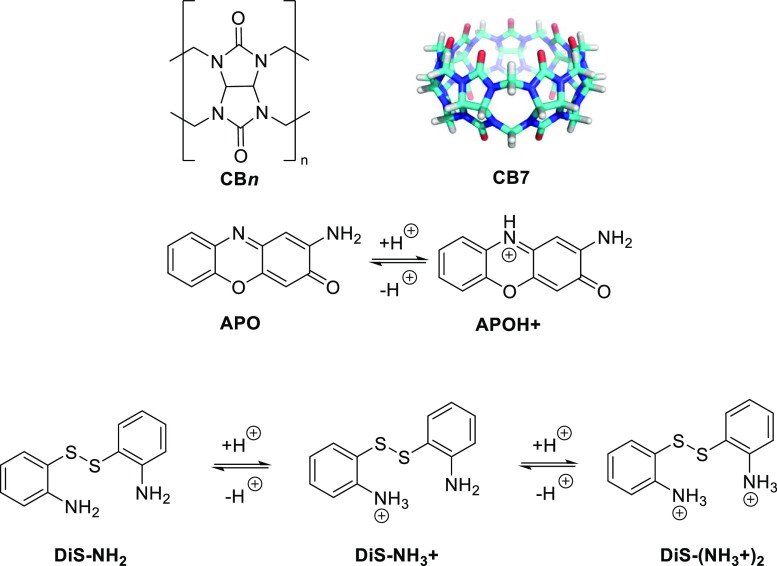
Chemical structures of **CB*n*** (*n* = 5–8), **APO**, and **DiS-NH**_**2**_ and their different protonation states.

The natural product **APO** presents a tricyclic structure with a primary amino group and morpholino ring, which are susceptible to be protonated, depending on soil acidity (Figure 1). Its production starts with the release of DIBOA (2,4-dihydroxy-(2*H*)-1,4-benzoxazin-3(4*H*)-one) by the plant, after which BOA (2-benzoxazolinone) is generated, and finally, **APO** is produced as a degradation compound of 2-aminophenol.^16^ This aminophenoxazinone shows promising inhibitory activity for use as a potential agrochemical, and its allelopathic interaction has been demonstrated in crop studies.^16^ However, the water solubility of this compound is extremely low (3.75 μM), thus limiting its application in an actual agrochemical setting.

The same challenge has been identified for **DiS-NH**_**2**_. This aminophenoxazinone mimic incorporates a disulfide bridge that connects two o*-*substituted aromatic rings. It exhibits a similar bioactive profile and agrochemical action to **APO** and has been extensively studied as an aminophenoxazinone model. The water solubility of **DiS-NH**_**2**_, although higher than that of the natural compound, is still too low (146.8 μM) for applications.

The protonated form of **APO** showed higher water solubility than the neutral form, thus making the acidification of the medium a principal option for enhancing the concentration of the natural product. However, the experimental p*K*_a_ value of **APO** is 2.9, which is slightly too acidic according to the Natural Resources Conservation Service of the United States Department of Agriculture (NRCS-US).^17^ Therefore, the protonation of neat **APO** in acidic formulations presents no viable route for subsequent agricultural application. Simulation of p*K*_a_ has been developed by employing *Major* Microspecies *Plugin* from Chemaxon to evaluate the percentage of protonated species according to the pH media (Figure S1).^18^ The p*K*_a_ values of **DiS-NH**_**2**_ also lie at around 2–3 (Figure S1), according to experimental titrations (p*K*_a_ = 2.1) and empirical speciation diagrams, which leads to the same challenge as for **APO**. On the other hand, cucurbiturils are well known to serve as “supramolecular acid substitutes” which can shift the p*K*_a_ value of the complexed guest molecule toward the more basic end.^7^ The encapsulation of **APO** and **DiS-NH**_**2**_ in cucurbiturils could therefore be a promising solution to make progress in the agricultural applications of aminophenoxazinones.

The intermolecular interactions between host and guest molecules, as well as size compatibility, are the two main factors that determine the binding affinity for host–guest complexation. As such, B3LYP/LanL2DZ DFT studies were performed to obtain the Gibbs free energy of the 1:1 complexes formed between the neutral analytes and four cucurbituril homologues (Figure S3a,b). According to Table S2, it has been revealed that the complexation of analytes (**APO** and **DiS-NH**_**2**_) present positive Δ*G* for all **CB*n***, specifying the lack of spontaneity. The cavity sizes of **CB5** and **CB6** (68 and 142 Å^3^, respectively) are too small to allow **APO** (174 Å^3^) and **DiS-NH**_**2**_ (214 Å^3^) (Figure S2) to be completely accommodated, although partial interactions remain possible.^19^ Furthermore, **CB7** (242 Å^3^) could be tight, so the interaction energy is still high to offer stabilization. In the case of **CB8** (367 Å^3^), it could be due to the lack of van der Waals interactions between the inner cavity and the guest compound.^20^

**Figure 2:**
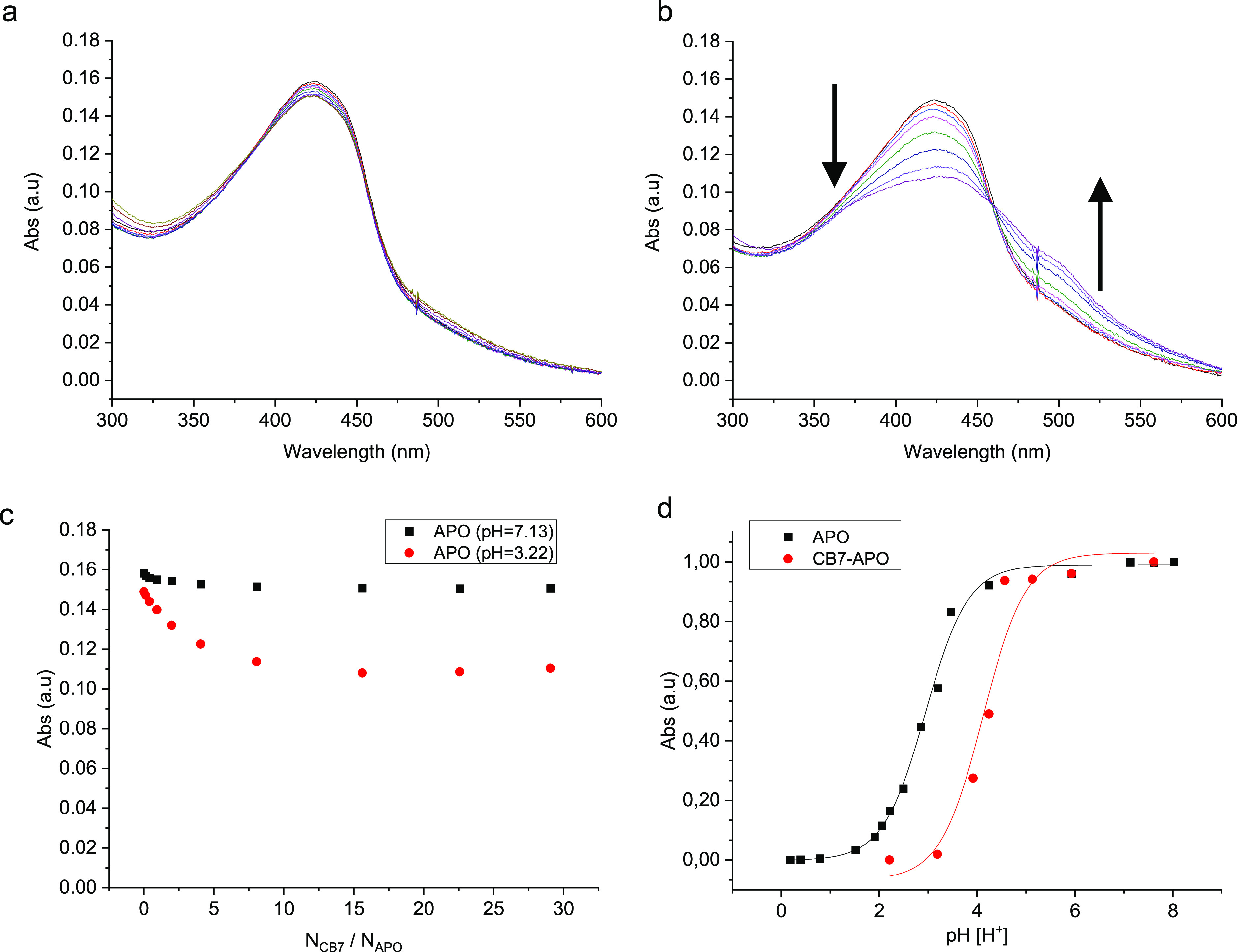
(a) UV–vis absorption spectral titration of **APO** (5 μM) with **CB7** (0–160 μM) at pH 7.13. (b) UV–vis absorption spectral titration of **APO** (5 μM) with **CB7** (0–160 μM) at pH 3.22. (c) Corresponding absorbance of **APO** at 434 nm with increasing *N*_**CB7**_/*N*_**APO**_ ratio in neutral and acidic solutions. (d) Normalized pH-dependent absorbance of the **APO** and **CB7·APO** (1:1) complex at 434 nm. The experiments have been developed in pure H_2_O (Milli-Q quality), 25 °C. Acidic media have been adjusted by adding HCl.

**Figure 3:**
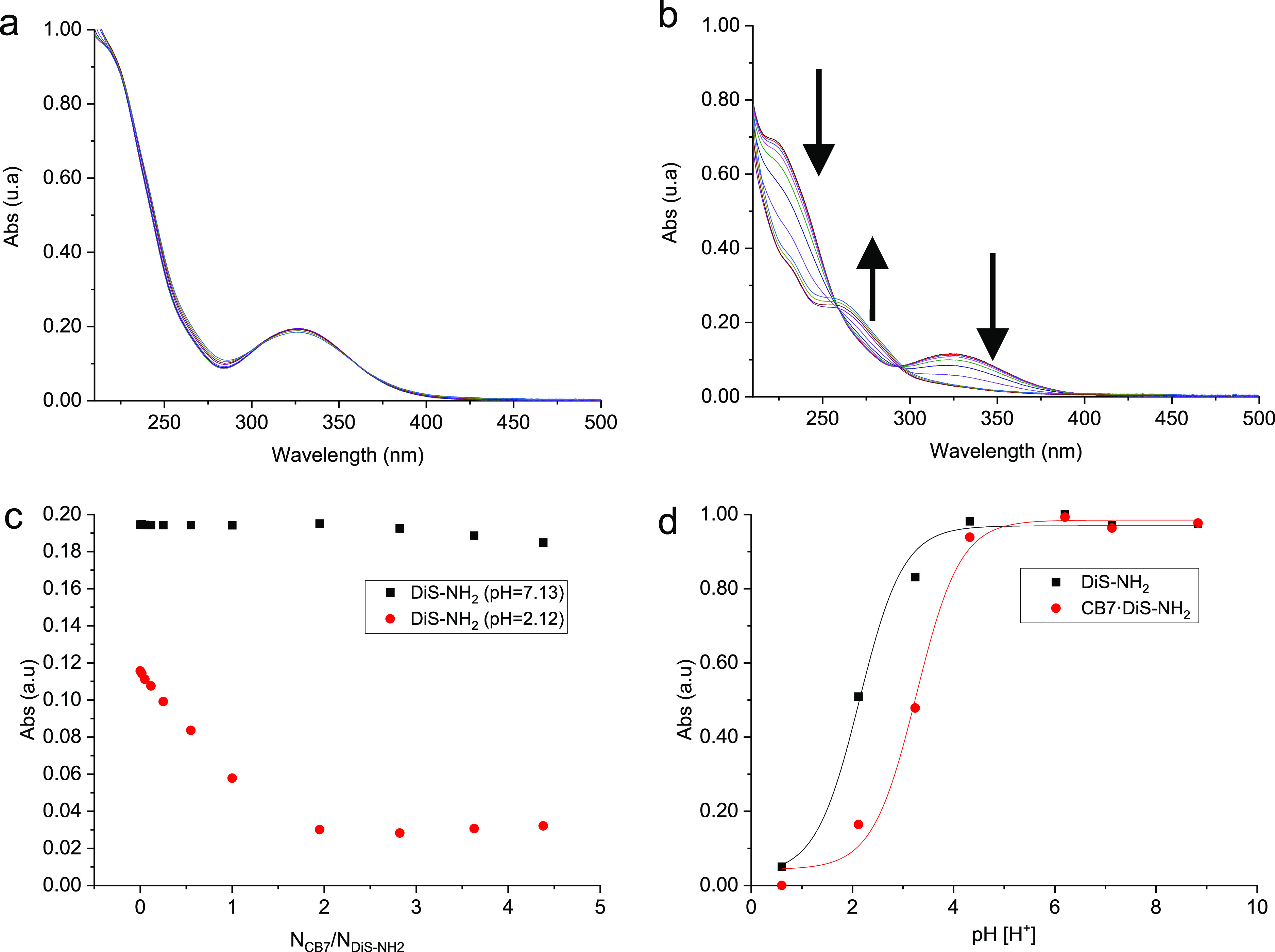
. (a) UV–vis absorption spectral titration of **DiS-NH**_**2**_ (40 μM) with **CB7** (0–180 μM) at pH 7.13. (b) UV–vis absorption spectral titration of **DiS-NH**_**2**_ (40 μM) with **CB7** (0–180 μM) at pH 2.12. (c) Corresponding absorbance of **DiS-NH**_**2**_ at 327 nm with increasing *N*_**CB7**_/*N*_**APO**_ ratio in neutral and acidic solutions. (d) Normalized pH-dependent absorbance of the **DiS-NH**_**2**_ and **CB7·DiS-NH**_**2**_ (1:1) complex at 327 nm. The experiments have been developed in pure H_2_O (Milli-Q quality), 25 °C. Acidic media have been adjusted by adding HCl.

Protonated molecules are well known to be stabilized by **CB*n***. We used DFT simulations to evaluate the feasibility of inclusion complex formation with protonated **APOH**^**+**^ and **DiS-NH**_**2**_ (**DiS-(NH**_**3**_^**+**^**)**_**2**_) and obtained sensible complex geometries (Figure S3c). Accordingly, titration experiments were carried out to determine the binding constants (*K*_a_). Complex formation was evaluated initially for both **APO** (5 μM) and **DiS-NH**_**2**_ (40 μM) in neutral solution, and the spectra of both guests show only minor variations after adding a large excess of **CB7**. (Figures 2a and 3a), indicating very weak host–guest interactions. However, the spectra of the guest molecules are dramatically changed when the titrations were performed in acidic media (Figures 2b and 3b). This revealed pronounced intermolecular interactions between **CB7** and the protonated guests **APOH**^**+**^, **DiS-NH**_**3**_^**+**^, and **DiS-(NH**_**3**_^**+**^**)**_**2**_.

Analysis at different pH values showed a marked modification of the p*K*_a_ value for the biomolecules after complexation with **CB7** (Figures 2d and 3d). The free **APO** has a p*K*_a_ value of 2.94 ± 0.30, whereas the complexed form **CB7·APOH**^**+**^ (1:1) exhibited an increase of more than one unit to 4.12 ± 0.15. In the case of **DiS-NH**_**2**_, the p*K*_a_ of the free guest is 2.14 ± 0.09, while the **CB7·DiS-NH**_**3**_^**+**^ (1:1)/**CB7·DiS-(NH**_**3**_^**+**^**)**_**2**_ (1:1) complexes exhibit a p*K*_a_ value of 3.26 ± 0.09. This means that complexation by **CB7** could allow these biomolecules to be applied directly in more kinds of crops as highly acidic solutions are no longer required. According to NRCS-US, if the acidity category has been changed for **APO** because of a higher p*K*_a_ value after complexation, this will allow it to be applied to different relevant crops, such as spring oats, winter oilseed rape, spring lupins, or potatoes. Holland et al.^21^ demonstrated that these crops have normal yield values with soil pH values of around 4.5.

While **APO** in the neutral state did not show significant interaction with **CB7**, the protonated form **APOH**^**+**^ is complexed by **CB7** with a moderate-to-high binding affinity of *K*_a_ = (1.85 ± 0.37) × 10^6^ M^–1^ as determined from optical titrations and fitting with a 1:1 binding model. Similarly, for **DiS-NH**_**2**_ and **CB7**, clear changes in the UV–vis spectra were observed only after protonation. The appearance and disappearance of the two isosbestic points upon the addition of **CB7** indicate the existence of more than one complexation state (see Figure 2b). First, they were estimated as *K*_obs_, but extended calculations have allowed us to determine which of the bands correspond with every protonated form. A titration experiment was performed at pH 2.12, where the main protonated species is **DiS-(NH**_**3**_^**+**^**)**_**2**_. Furthermore, according to DFT simulations, there is a hypsochromic effect due to double protonation of the molecule, and the band observed at 255.0 nm is shifted to 221.3 nm. The shielding of this band was followed to obtain the binding constant of **DiS-(NH**_**3**_^**+**^**)**_**2**_ with **CB7**. On the other hand, a titration experiment at pH 3.27 was carried out to obtain the binding constant of monoprotonated disulfide. The main species present at this pH value is **DiS-NH**_**3**_^**+**^ (Figure S1), and the most affected band due to complexation is 326.7 nm. This agrees with theoretical results, previous experiments carried out and shown in Figure S5, and the UV expected after **CB7** complexation with protonated species of **DiS-NH**_**2**_ (Figure S6).

The low water solubility and low synthetic yield of **CB8** make it less ideal for our purposes. In contrast, despite the low water solubility of **CB6**, its economic availability at a larger scale motivated its use in further studies. Thus, studies on the modulation of p*K*_a_ were also carried out with **CB6**, but no indications for complexation were observed, unquestionably due to its small cavity size (Figure S4). As such, only the inclusion complexes generated with **CB7** were able to modify the physicochemical properties (p*K*_a_). The UV–vis absorption spectra of **APO** and **APOH**^**+**^ did not show any spectral shift, which is in contrast to the behavior observed for **APOH**^**+**^ complexed with **CB7** where a bathochromic shift from λ_max_ = 436.0 nm to λ_max_ = 443.3 nm was observed when complexation starts, with one isosbestic point at λ_max_ = 459.0 nm related to the new band that appears at λ_max_ = 498.0 nm. In the case of **DiS-NH**_**2**_, a large difference in the absorption spectra was observed between the doubly charged and the monocharged species according to the simulation (Figure S5d). With the monocharged **DiS-NH**_**2**_, both the main band at ∼200 nm and the band at ∼400 nm almost vanished after the second protonation, while the band at ∼260 nm reached the highest, which is in agreement with the experimental observations shown in Figures 2b. When **DiS-(NH**_**3**_^**+**^**)** or **DiS-(NH**_**3**_^**+**^**)**_**2**_ is complexed by **CB7**, the band at ∼200 nm is energetically decreased, while the peak at ∼260 nm starts to appear.

The calculated electronic absorption spectrum of **APOH**^**+**^ (Figure S5a) shows the same bathochromic shift behavior after complexation with **CB7** as that observed experimentally. According to the B3LYP/LanL2DZ DFT Mulliken charge calculation, this can be explained by a modification of the electronic density on the different groups after encapsulation (Table S3). Energy-transfer processes are common in the complexation of macrocycles such as cucurbiturils and cyclodextrins.^22−26^ It is found that there is a charge transfer from **CB7** toward the aromatic ring of **APOH**^**+**^ (H5–H8) and the carbonyl group (O12), which may be responsible for the emergence of the new band at λ_max_ = 498.0 nm. The UV spectral changes for **DiS-NH**_**3**_^**+**^ and **DiS-(NH**_**3**_^**+**^**)**_**2**_ could also be explained similarly due to strong modification of the polarity of the molecules after complexation. For example, Figure S5g shows a zero net dipolar moment for **DiS-(NH**_**3**_^**+**^**)**_**2**_, which increases to 5.42 D after encapsulation in **CB7**. We also compared the stability of the complexes formed by monoprotonated and doubly protonated **DiS-NH**_**2**_ in **CB7**. It was revealed that the doubly charged **DiS-(NH**_**3**_^**+**^**)**_**2**_ is more favorable by **CB7**, with both ammonium ions stabilized at the carbonyl portals and the disulfide bond residing inside the cavity of **CB7** (Figure S5h, right).

NMR analysis also demonstrated the formation of the **APOH**^**+**^**·CB7** complex, as revealed by the significant change in the chemical shifts in the ^1^H NMR spectrum. This study was possible due to the sufficient water solubility after pH modulation. ^1^H NMR spectroscopy was conducted in D_2_O at pD 2.6 (adjusted with DCl), and the complexation-induced chemical shifts were followed (Figure S7). Specifically, all aromatic ring protons (H6–H9) experienced a pronounced up-field shift due to re-location inside the non-polar cavity. In addition, H4 showed a significant downfield shift from 6.59 to 7.13 ppm, which is an indication of its position at the **CB7** carbonyl rim. In the case of **DiS-(NH**_**3**_^**+**^**)**_**2**_, the experiment was conducted at pD 1.9 to ensure that the doubly protonated **DiS-(NH**_**3**_^**+**^**)**_**2**_ was the main species. All signals were downfield-shifted, in contrast to the benzene moiety of **APOH**^**+**^, for which the signals were shifted upfield. Figure S3c shows that the carbonyl group and the amino group are outside of the toroid **CB7** shape, showing more interactions with the solvents in contact with the benzyl ring. This agrees with NMR signals because of bigger shifting for H6–H9 protons in comparison with H1 and H4 in the outside part. Taking a look closer to the arrangement of guested **APOH +** in **CB7**, it is observed that it is not fully perpendicular and the proton close to carbonyl group (H4) is closer to the carbonyl portal than the proton close to the amino group (H1). This causes the highest shift (Δδ = ±0.5 ppm) in signal H4 observed in Figure S7. In addition, the resolution of the signals was reduced, but the intensity increased due to the higher solubility of the complex in D_2_O. All the experiments shown in Figure S7 were performed at 1 mg/mL, and the same NMR parameters were applied. In this case, the intensity of the signal shows the highest availability of the complex **DiS-(NH**_**3**_^**+**^**)**_**2**_**·CB7** in solution in comparison to the uncomplexed form. According to Figure S5h, the DFT simulations predict a lower-energy structure for the symmetric complex with the disulfide bridge in the inner cavity of **CB7**, where the NH_3_^+^ groups establish hydrogen bonds with the carbonyl groups at both portals. The lack of resolution is explained by different microenvironments of every ring of **DiS-(NH**_**3**_^**+**^**)**_**2**_. This lack of symmetry generates the broadness, due to different interactions with every part of **CB7**. H4 and H6 are the furthest from interactions with the macrocycle, according to the model observed in Figure S3c, so this impacts in a lower Δδ, as is observed in Figure S7.

The stability of the **CB7·APOH**^**+**^ complex was evaluated by performing kinetic experiments at pH 2.12 (Figure S8 left). Absorbance data were collected at λ_max_ for 90 min, and almost no change was noted, demonstrating that the 1:1 complex remained stable in solution for at least this period of time. Similar results were observed with **DiS-NH**_**2**_ at pH 1.5 (Figure S8 right), thus confirming the stability of the 1:1 complex in acidic media. The mass spectra recorded after the kinetic study confirmed the complex formation, showing [**CB7·APOH**^**+**^**·H**^**+**^]^2+^: 688.2111 amo (calculated: 688.2080 amo) and [**CB7·DiS-(NH**_**3**_^**+**^**)**_**2**_]^2+^: 706.2027 amo (calculated: 706.2015 amo). Both free analytes were also tested for stability at pH 2.12 and pH 1.5, respectively, and showed great stabilities in acid. In contrast, the stability of the analytes in basic media is in general much worse, even with the addition of **CB7** or **CB6**. It was found that around 35% of **APO** was degraded after a kinetic period of 90 min (see the Supporting Information). The addition of 1 equiv. of **CB7** only slowed down this process to around 30%, an insignificant difference. Meanwhile, the addition of **CB6** made the degradation happen even faster. The same phenomenon is also observed in the case of **DiS-NH**_**2**_ for which **CB7** reduced the degradation slightly, while **CB6** accelerated it, likely due to a supramolecular catalytic effect (Figure S9).

**Figure 4:**
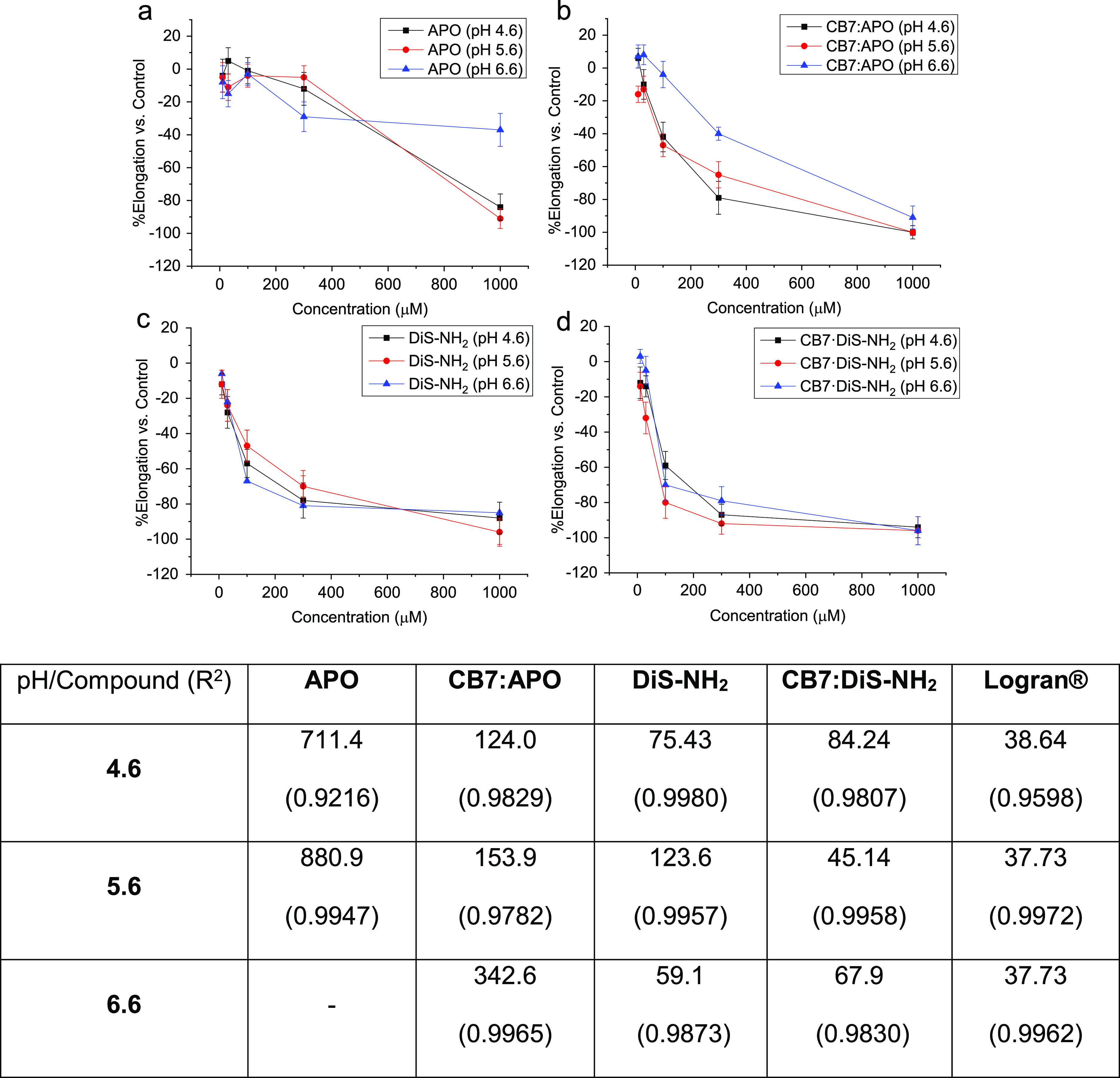
Results of in vitro bioassays with wheat coleoptiles. (a) **APO**, (b) **APO·CB7**, (c) **DiS-NH**_**2**_, and (d) **DiS-NH**_**2**_**·CB7** were tested at different pH values. Table: IC_50_ values for the complexes, the free compounds, and a positive control.

**CB7** complexes were evaluated in vitro to test their efficacy as potential bioherbicides with enhanced physicochemical properties. **APO** and **DiS-NH**_2_ have been previously studied as lead compounds for agrochemical synthesis with some success.^5,14,16^ In detail, we carried out experiments with etiolated wheat coleoptile, using high concentrations of undifferentiated plant cells, at different pH values. These evaluations showed that the complexes exhibited different bioactivities depending on the acidity, thus supporting our previous studies (Figure S10). Figure 4 presents plots and *IC*_50_ values to compare the bioactivity profiles, and it can be clearly observed that **CB7** encapsulation enhances the biological activity. Thus, in the case of **APO**, the *IC*_50_ is reduced more than five times when the bioactive compound is complexed by cucurbituril in acidic media. This is also clear from Figure 3a,b, where it can be seen how the percentage elongation decreases at the same concentration after encapsulation. For example, 300 μM showed 80% inhibition when **APO** is encapsulated at pH 4.6, in contrast with <10% inhibition displayed by free **APO**. In the case of IC_50_, at pH 6.6, **APO·CB7** exhibits a value of 343 mM at the higher concentration, whereas the free compound is essentially inactive. Acidification of the medium results in an increase in bioactivity. Thus, in the case of the free compound, the water solubility is enhanced after protonation, and this explains the increase in activity at 1000 μM. In the case of **APO·CB7**, complexation allows the inhibition of elongation at lower concentrations. For example, the increase in bioactivity at 300 μM is directly dependent on pH and therefore directly dependent on the complexation degree due to the large number of protonated species available in the solution.

In addition to the inhibition of elongation, the appearance of the coleoptiles was also modified by application of the bioherbicides. Figure S11a shows how **CB7** encapsulation of **APO** at pH 4.6 causes an orange staining, which is not seen in the free compound bioassay. This could be explained by the higher solubility and transport of the compound after complexation with **CB7**. The bioassay performed at pH 5.6 (Figure S11c,d) does not show any staining due to the small quantities of the charged **APO** species present in the solution test (according to the p*K*_a_ curve in Figure 2a) due to the fact that they are encapsulated by **CB7**, thereby enhancing their physicochemical properties.

The inhibition values for **DiS-NH**_**2**_ are also significant, although lower than those for **APO**, due to the intrinsic water solubility. This compound is already known to be an interesting agrochemical lead, and **CB7** can improve its activity further, as shown in Figure 3d. A comparison of the bioactivity results at 300 μM shows that the elongation percentage is reduced from around 60% to around 80% at lower pH (pH 4.6 and 5.6). At 100 μM, the results are more pronounced upon complexation. Thus, as can be seen from Figure S10, **CB7** presents a significant activity at 1000 μM, but the bioactivity results at lower concentration can only be explained by a complexation process.

The results obtained at pH 4.6 and 5.6 are similar for **APO·CB7** and **DiS-NH**_**2**_**·CB7** to the positive control Logran. The main component of this control is triasulfuron, a halogenated and highly environmentally persistent herbicide. In contrast, aminophenoxazinones are natural compounds produced by plants as a part of their own defensive system; therefore, their assimilation and degradation by other plants and microorganisms after application are in any case more environmentally friendly.

In conclusion, we have reported the supramolecular host–guest interaction and p*K*_a_ modifications of aminophenoxazinones and their disulfide mimics by **CB7** for the first time. UV–vis absorption spectral titrations showed moderate-to-high binding affinities, with values >10^6^ M^–1^ and modification of their p*K*_a_ values by more than one unit for every compound. NMR spectroscopy confirmed the change in the chemical microenvironment due to **CB7** complexation by way of a change in the chemical shifts. The stability of the compounds after complexation was analyzed at different pH values, and it was found that the addition of **CB7** to **APO** and **DiS-NH**_**2**_ in a 1:1 ratio prevents degradation, whereas the smaller homologue **CB6** promotes it. This suggests the formation of complexes with the protonated species. In vitro studies with wheat ethiolated coleoptiles demonstrate that **CB7** is able to enhance the bioactivity of the guest molecules by modifying its physicochemical properties. In addition, as a result of p*K*_a_ modification, **APO** and **DiS-NH**_**2**_ could be applied to protect a larger range of crops as they can now be used in a new soil acidity range. These findings provide a new strategy to apply aminophenoxazinones and their mimics to plant systems using the supramolecular organic-solvent-free method, thus resulting in a new family of green agrochemicals.

## Supporting Information Available

Theoretical p*K*_a_ diagrams for **DiS-NH**_**2**_ and **APO**; amount and content of synthesized **CB*n***; results of B3LYP/LanL2DZ DFT studies; structure of **DiS-NH**_**2**_, APO, and **CB7**; optimized structures of neutral and protonated compounds with all the **CB*n***; Mulliken charges distribution; p*K*_a_ curve of APO with **CB7**; calculated and experimental UV–vis spectra of the **APOH**^**+**^ and **APOH**^**+**^**·CB7** 1:1 complex; dipolar moment representation of the complexes; calculated and experimental UV–vis spectra of **DiS-NH**_**3**_^**+**^ and **DiS-(NH**_**3**_^**+**^**)**_**2**_ and their **CB7** complexes; titration experiment of **DiS-NH**_**3**_^**+**^ and **DiS-(NH**_**3**_^**+**^**)**_**2**_ with **CB7** at different pH values; NMR spectra of **APOH**^**+**^, **APOH**^**+**^**·CB7**, **DiS-(NH**_**3**_^**+**^**)**_**2**_, and **DiS-(NH**_**3**_^**+**^**)**_**2**_**·CB7**; stability experiment of complexes in acidic media; kinetic study of complexes in basic media; NMR spectra of complexes in basic media; in vitro results of wheat coleoptile bioassay; and images of wheat coleoptile after 24 h of complexes treatment (PDF)
