# Supplementary material for: Stability and pKa Modulation of Aminophenoxazinones and Their Disulfide Mimics by Host–Guest Interaction with Cucurbit[7]uril. Direct Applications in Agrochemical Wheat Models
Source: J Agric Food Chem. 2022 Dec 22;71(1):480–7. doi: 10.1021/acs.jafc.2c06373 (PMC9837879; doi:10.1021/acs.jafc.2c06373)
Supplement: Supplementary file 1 — jf2c06373_si_001.pdf [file jf2c06373_si_001.pdf]

Supporting Information

Stability and pK_a Modulation of Aminophenoxazinones and their Disulfide Mimics by Host–Guest Interaction with Cucurbit[7]uril. Direct applications in agrochemical wheat models

Francisco J.R. Mejías[1,2], Suhang He[2], Rosa M. Varela[1], José M.G. Molinillo[1], Andrea Barba-Bon[2], Werner M. Nau[2]*, Francisco A. Macías[1]*

[1] Allelopathy Group, Department of Organic Chemistry, Institute of Biomolecules (INBIO), University of Cádiz, República Saharaui 7, 11510 Puerto Real Cádiz, Spain.

[2] Department of Life Sciences and Chemistry, Jacobs University Bremen, Campus Ring 1, 28759 Bremen, Germany.

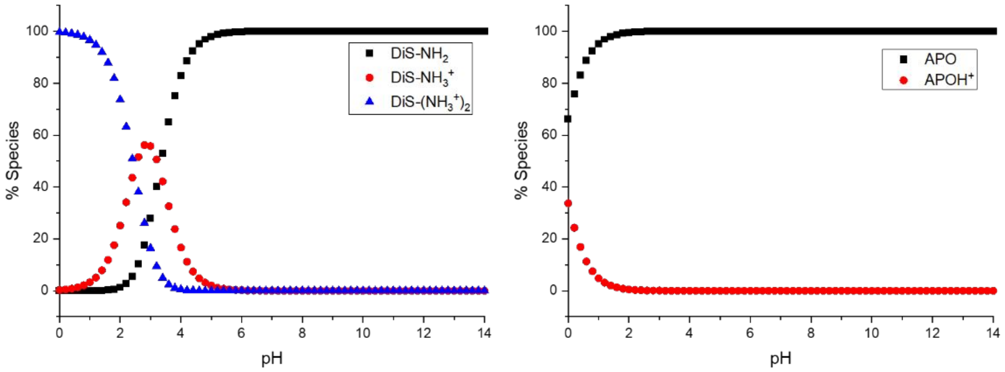

Figure S1. Theoretical DiS-NH_2 and APO microspecies distributions to be compared with the experimental values obtained. In case of DiS-NH_2, theoretical pKa is (3.2-3.4), while experimental value is 2.1. Theoretical pKa value for APO is not in the range from 0-14, while experimental value is 2.9.

Table S1. Data of the obtained from synthesis of CB*n*.

CBn	CB5	CB6	CB7	CB8
Amount (g)	0.72	20.66	7.85	0.88
Content (%)	< 70%	86 ± 2	76 ± 3	86 ± 3

Table S2. Results of B3LYP/LanL2DZ DFT studies.

$$\Delta G = G_{CB\cdot Guest} - (G_{CB} + G_{Guest})$$

$$\Delta H = H_{CB\cdot Guest} - (H_{CB} + H_{Guest})$$

Compound	G (kJ/mol)	H (kJ/mol)	ΔG (kJ/mol)	ΔH (kJ/mol)
CB5	-7.90E+06	-7.90E+06	n.a.	
CB6	-9.48E+06	-9.48E+06		
CB7	-1.11E+07	-1.11E+07		
CB8	-1.26E+07	-1.26E+07		
APO	-1.90E+06	-1.89E+06		
APOH+	-1.90E+06	-1.90E+06		
CB5·APO	-9.79E+06	-9.79E+06	402.993246	328.707349
CB6·APO	-1.14E+07	-1.14E+07	170.108770	107.842413
CB7·APO	-1.30E+07	-1.29E+07	43.662065	6.821049
CB8·APO	-1.45E+07	-1.45E+07	49.057467	-8.204688
CB7·APOH+	-1.30E+07	-1.30E+07	-0.711510	-39.051687

Compound	G (kJ/mol)	H (kJ/mol)	ΔG (kJ/mol)	ΔH (kJ/mol)
CB5	-7.90E+06	-7.90E+06	n.a.	
CB6	-9.48E+06	-9.48E+06		
CB7	-1.11E+07	-1.11E+07		
CB8	-1.26E+07	-1.26E+07		
DiS-NH_2	-1.56E+06	-1.56E+06		
CB5·DiS-NH_2	-9.46E+06	-9.45E+06	830.0229445	726.727898
CB6·DiS-NH_2	-1.10E+07	-1.10E+07	237.471224	157.464362
CB7·DiS-NH_2	-1.26E+07	-1.26E+07	89.94963	25.506732
CB8·DiS-NH_2	-1.42E+07	-1.42E+07	57.929032	1.801093
CB7·DiS-NH_3^+	-1.26E+07	-1.26E+07	37.581407	-17.420193
CB7·DiS-$(NH_3^+)_2$	-1.26E+07	-1.26E+07	-15.1097525	-85.961495

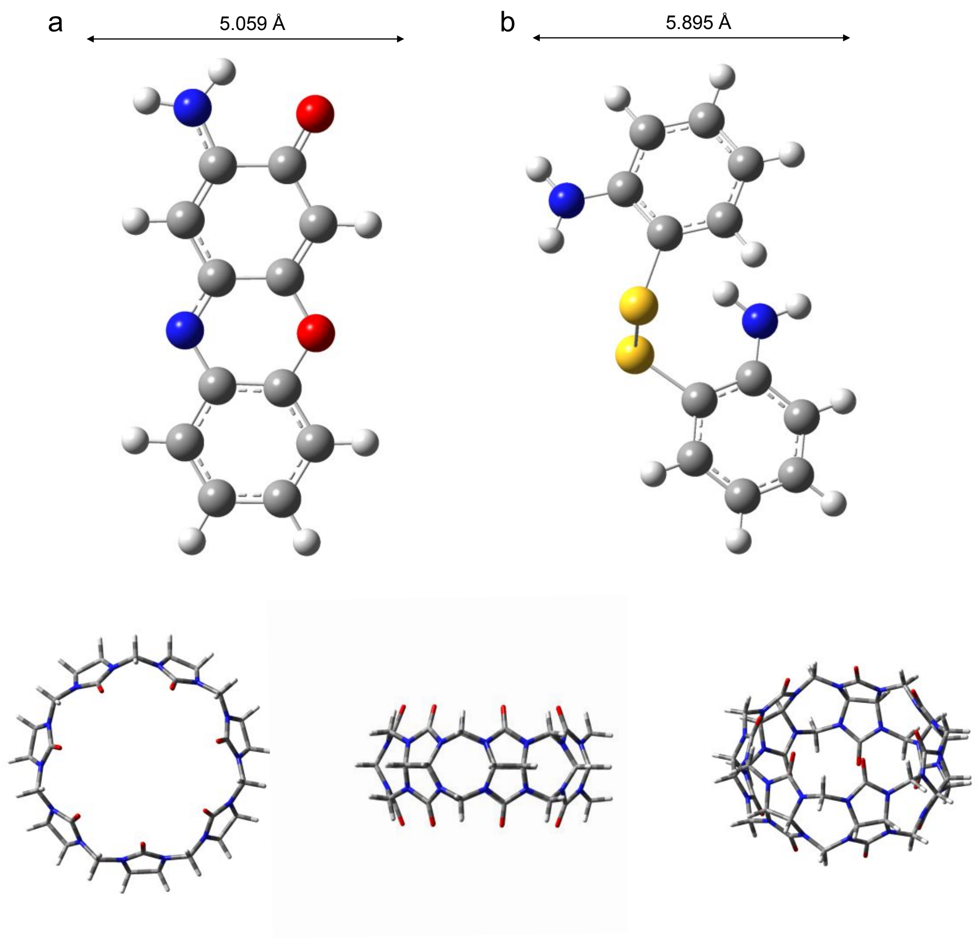

Figure S2. (Top) Structure, size and diameter of (a) **APO** and (b) **DiS-NH$_2$**. Calculated applying B3LYP/LanL2DZ DFT studies. Grey – C, White – H, Blue – N, Red – O, Yellow – S. (Bottom) Structure and shape of **CB7** from different perspectives.

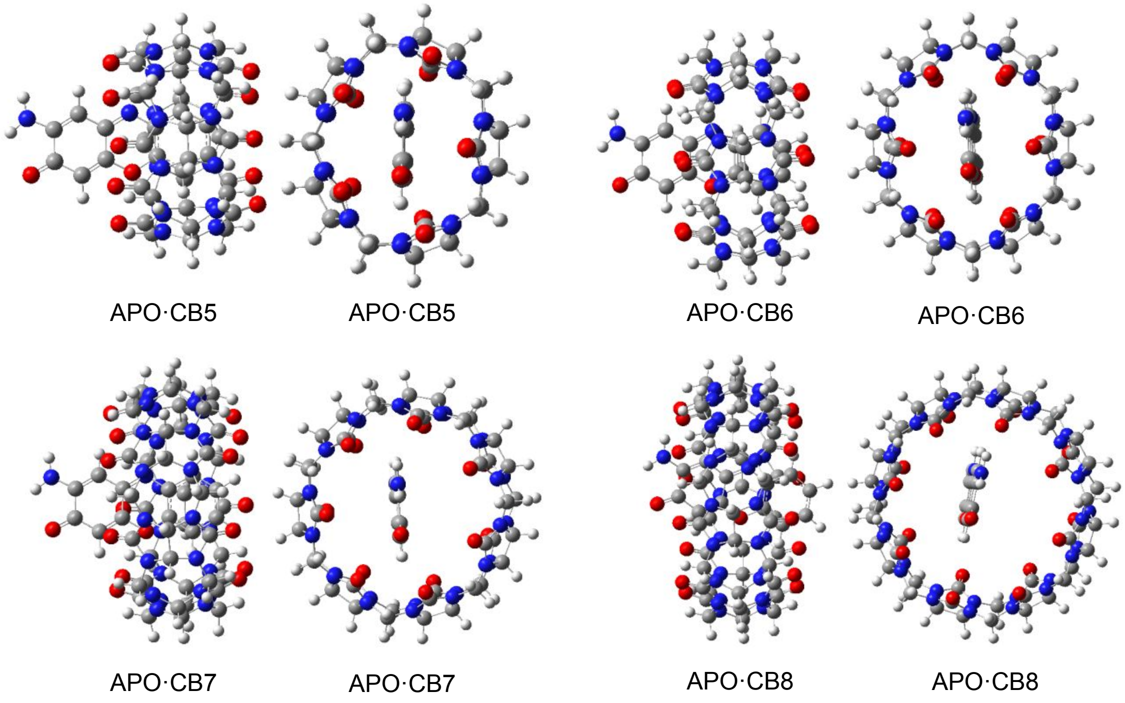

Figure S3a. Optimized structures of **APO·CB5**, **APO·CB6**, **APO·CB7** and **APO·CB8**. They have been calculated applying B3LYP/LanL2DZ scrf=(cpcm,solvent=water) DFT studies. Grey – C, White – H, Blue – N, Red – O, Yellow – S.

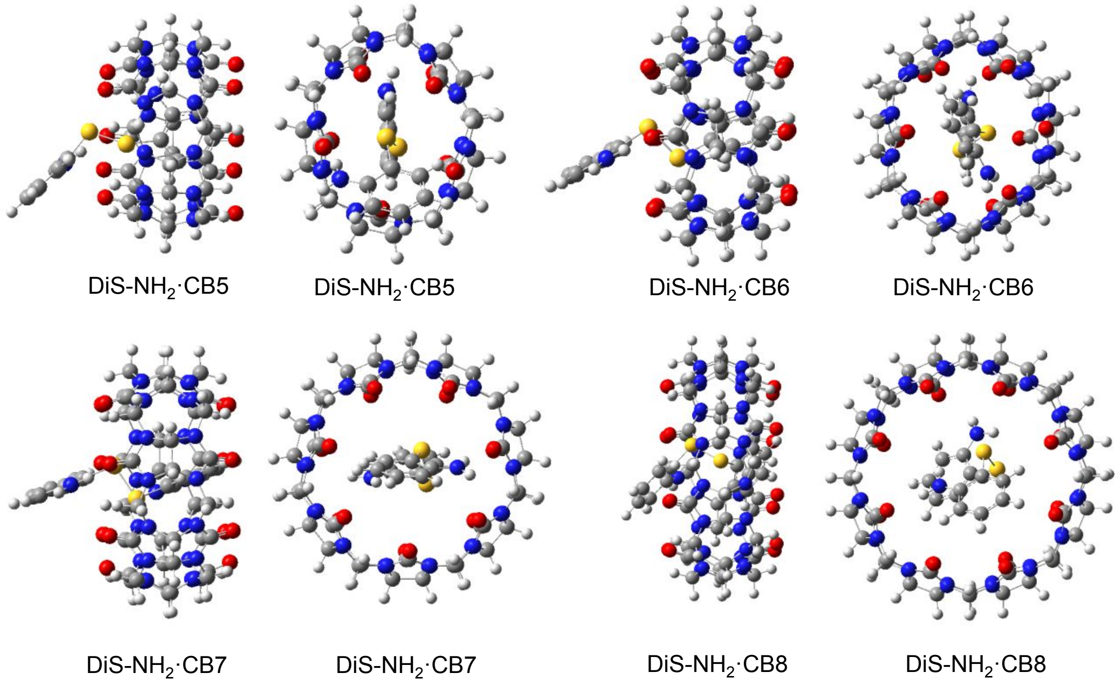

Figure S3b. Optimized structures of **DiS-NH_2·CB5**, **DiS-NH_2·CB6**, **DiS-NH_2·CB7** and **DiS-NH_2·CB8**. They have been calculated applying B3LYP/LanL2DZ scrf=(cpcm,solvent=water) DFT studies. Grey – C, White – H, Blue – N, Red – O, Yellow – S.

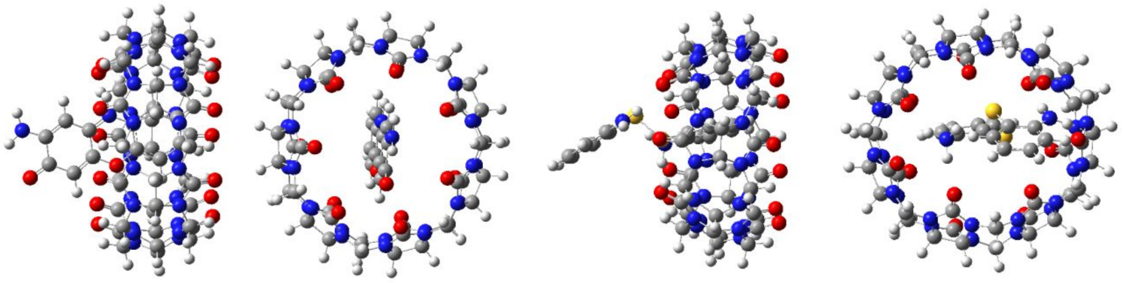

APOH^{+}·CB7 APOH^{+}·CB7 DiS-NH$_3^{+}$·CB7 DiS-NH$_3^{+}$·CB7

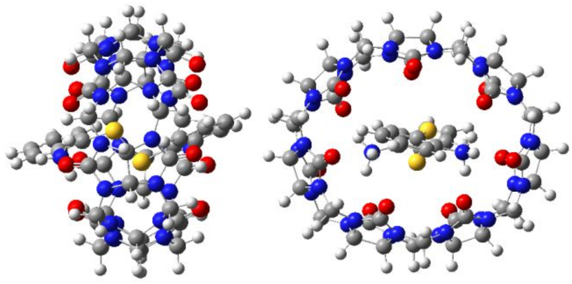

DiS-(NH$_3^{+}$)$_2$·CB7 DiS-(NH$_3^{+}$)$_2$·CB7

Figure S3c. Optimized structures of **APOH^{+}·CB7**, **DiS-NH$_3^{+}$·CB7** and **DiS-(NH$_3$)$^{+}$·CB7**. They have been calculated applying B3LYP/LanL2DZ scrf=(cpcm,solvent=water) DFT studies. Grey – C, White – H, Blue – N, Red – O, Yellow – S.

Table S3. Mulliken charges distribution per atom.

APOH$^+$				APOH$^+$·CB7			
Position/ Atom	Charge	Position/ Hydrogen	Charge	Position/ Atom	Charge	Position/ Hydrogen	Charge
C1	-0.121	H1	0.185	C1	-0.111	H1	0.197
C2	0.331	-	-	C2	0.322	-	-
C3	0.271	-	-	C3	0.264	-	-
C4	-0.145	H4	0.221	C4	-0.116	H4	0.245
C4a	0.334	-	-	C4a	0.336	-	-
C5a	0.266	-	-	C5a	0.265	-	-
C6	-0.116	H6	0.200	C6	-0.147	H6	0.172
C7	-0.112	H7	0.186	C7	-0.125	H7	0.206
C8	-0.119	H8	0.183	C8	-0.141	H8	0.248
C9	-0.121	H9	0.177	C9	-0.154	H9	0.203
C9a	0.357	-	-	C9a	0.365	-	-
C10a	0.405	-	-	C10a	0.411	-	-
O5	-0.559	-	-	O5	-0.570	-	-
N10	-0.833	H13	0.368	N10	-0.860	H13	0.422
N11	-0.738	H15, H16	0.370, 0.396	N11	-0.761	H15, H16	0.347, 0.372
O12	-0.385	-	-	O12	-0.431	-	-

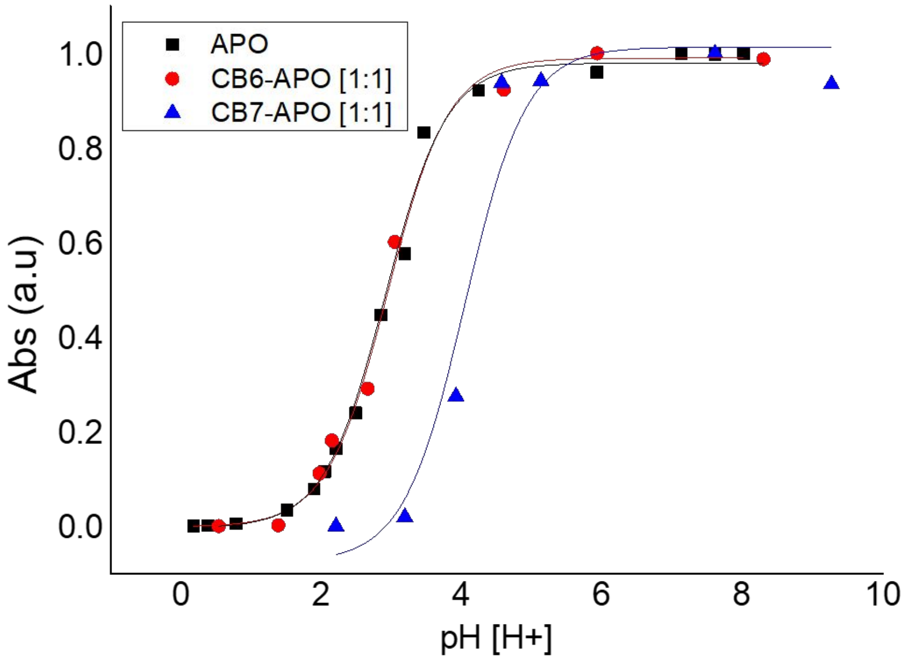

Figure S4. pKa curves of **APO** (5 μM) with **CB7** (160 μM) and **CB6** (16 μM) to confirm the lack of complex formation does not affect to pK_a values. The experiments have been developed in pure H_2O (mili-Q quality), 25ºC. Acid media have been adjusted by adding HCl.

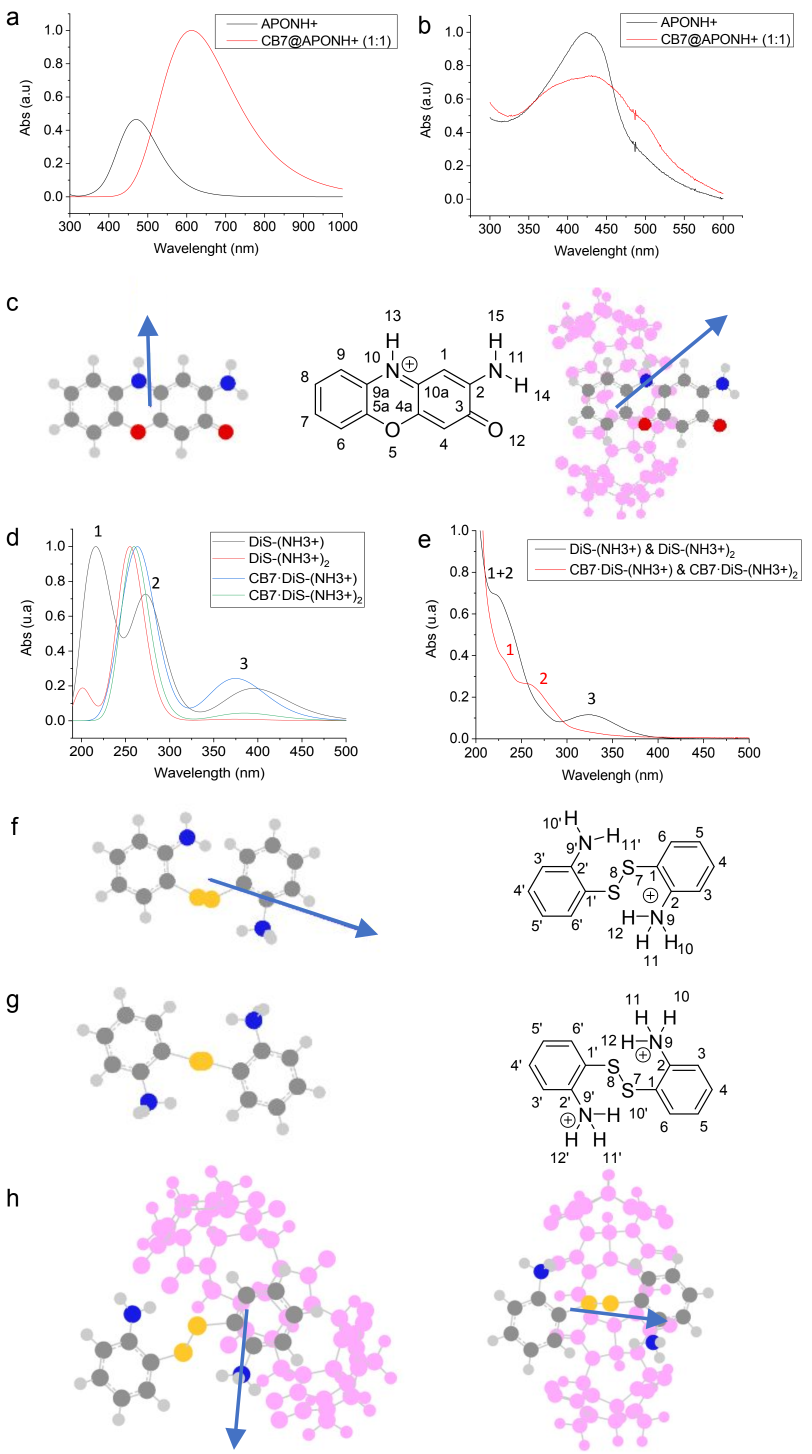

Figure S5. (a) Calculated and (b) experimental UV-Vis spectra of **APOH⁺** and **APOH⁺·CB7** 1:1 complex. The experiments have been developed in pure H_2O (mili-Q quality), 25ºC. Acid media have been adjusted by adding HCl. (c) Chemical representation and dipolar moments of **APOH⁺** and **APOH⁺·CB7**. (d) Calculated and (e) experimental UV-Vis spectra of **DiS-NH$_3^+$**, **DiS-(NH$_3^+$)$_2$** and their **CB7** complexes. The experiments have been developed in pure H_2O (mili-Q quality), 25ºC. Acid media have been adjusted by adding HCl. (f) Representation and dipolar moment of **DiS-NH$_3^+$**. (g) Representation and dipolar moment of **DiS-(NH$_3^+$)$_2$**. (h) Dipolar moments of **DiS-NH$_3^+$·CB7** (left) and **DiS-(NH$_3^+$)$_2$·CB7** (right).

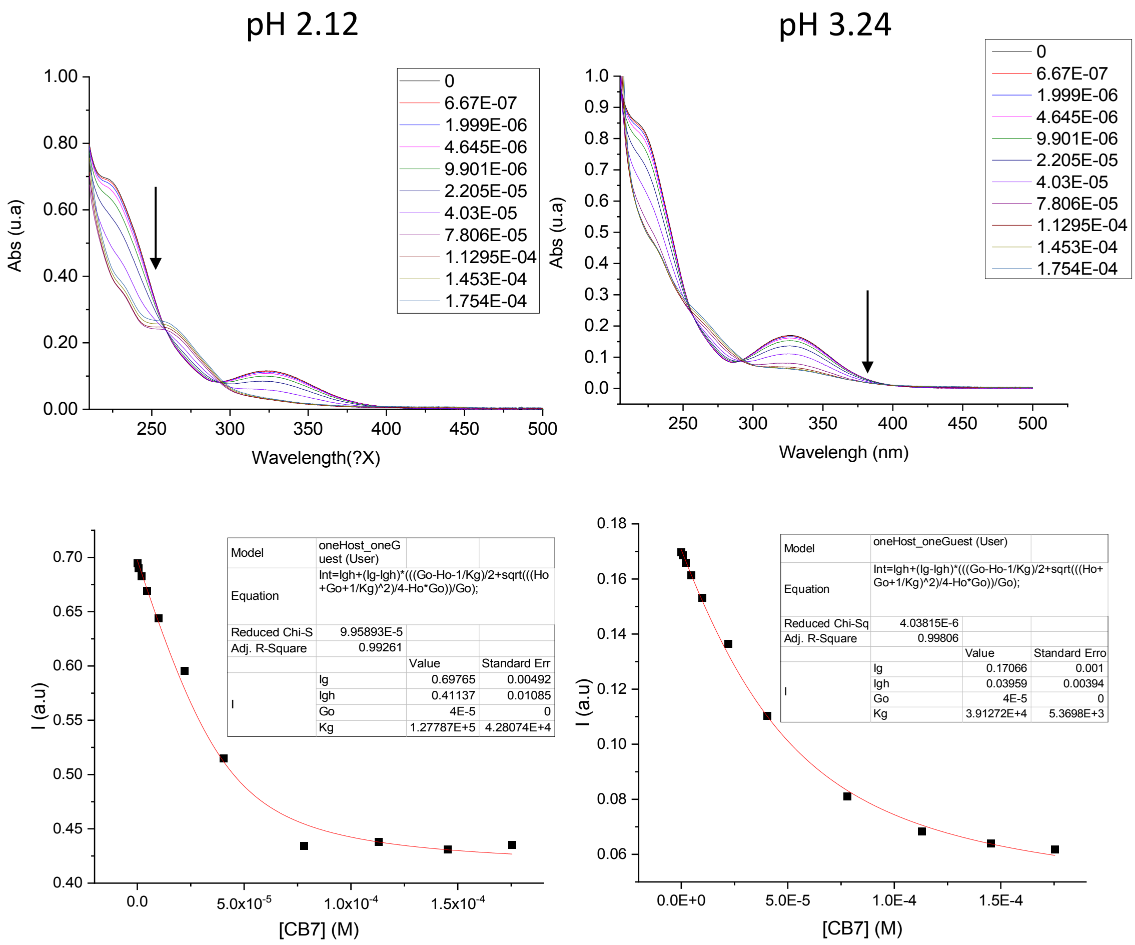

Figure S6. (Top left) Titration experiment at pH 2.12 of **CB7** (0-175 μM) with **DiS-(NH$_3^+$)$_2$** (40 μM) to calculate the binding constant. (Bottom left) Calculation of binding constant of **CB7** and **DiS-(NH$_3^+$)$_2$** with the intensity variation of 221.3 nm band. (Top right) Titration experiment at pH 3.34 of **CB7** (0-175 μM) and **DiS-NH$_3^+$** (40 μM) to calculate the binding constant. (Bottom right) Calculation of binding constant of **CB7** and **DiS-NH$_3^+$** with the intensity variation of 326.7 nm band.

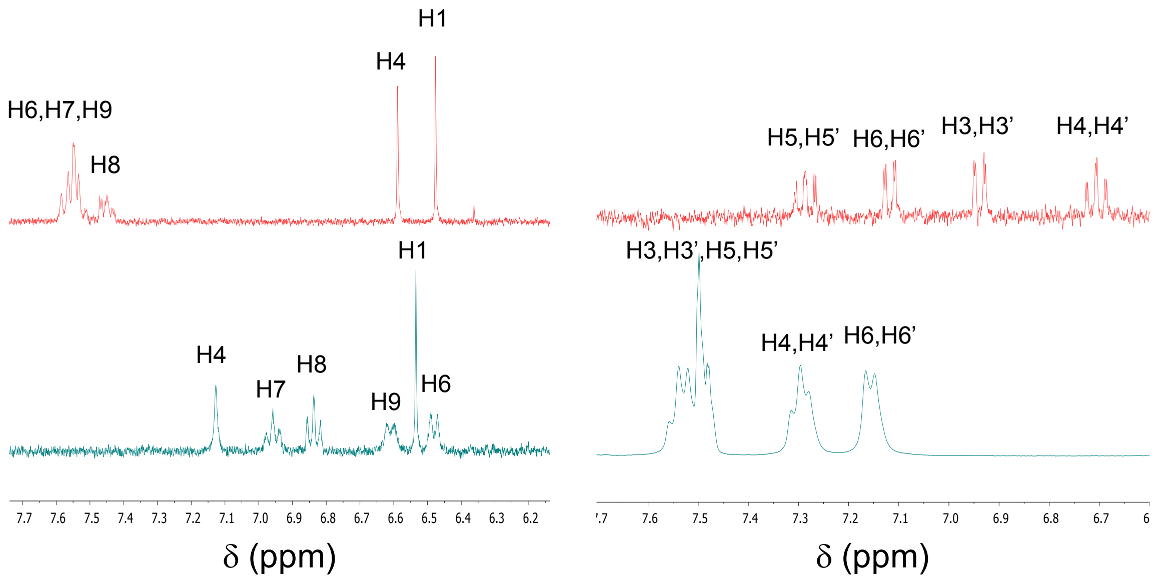

Figure S7. ^{1}H-NMR spectra of **APOH⁺** (top left) and **APOH⁺·CB7** (bottom left) in acidic D_2O (pD 2.6). ^{1}H-NMR spectra of **DiS-(NH$_3^+$)$_2$** (top right) and **DiS-(NH$_3^+$)$_2$·CB7** (bottom right) in acidic D_2O (pD 1.9). Both experiments have been carried out at 25ºC, and acid media have been adjusted by adding DCl.

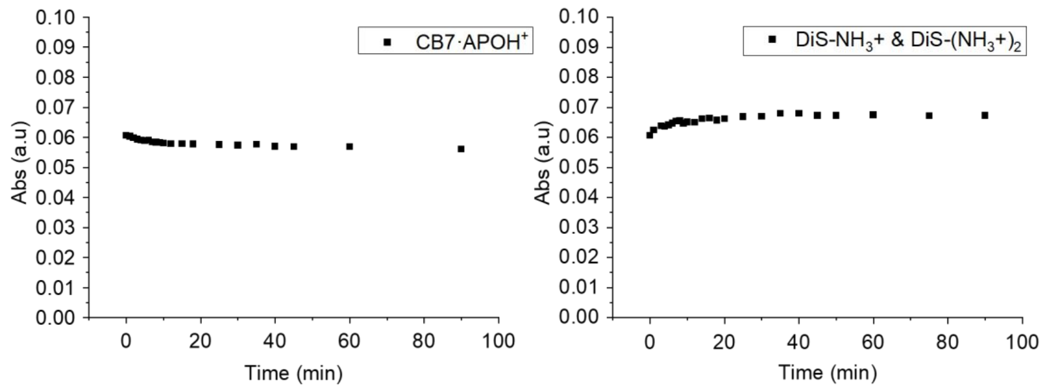

Figure S8. Stability of the complexes formed of CB7 and the protonated **APO** (5 μM, left) and **DiS-NH₂** (40 μM, right) in acid media (pH 2.12). The experiments have been developed in pure H_2O (mili-Q quality), 25ºC. Acid media have been adjusted by adding HCl.

Complex formation between **APO** and **CB7** was not significant under neutral conditions and was therefore studied in a basic medium (pH 10.0). Continuous exposure to a basic pH resulted in a modification of the absorbance irrespective of the **CB7** concentration added. The chemical transformation of **APO** was monitored by ^{1}H NMR spectroscopy (Figure S8) and it was found that 2-aminophenolate is regenerated when a strongly basic medium is used. It was found that around 35% of **APO** is degraded in 90 min and that the addition of **CB7** slowed this process down. Thus, after addition of one equivalent of **CB7**, degradation was reduced to around 30%, whereas two equivalents reduced this further to around 21%. These results indicate that although the addition of **CB7** to the **APO**

solution did not cause significant changes with regard to the previous spectroscopic titration, complexation still occurs and **CB7** protects **APO** against decomposition to some extent. In contrast, the use of **CB6** promoted the degradation of **APO**, irrespective of the amount applied. This suggests that the stabilization of **APO** is due to the formation of inclusion complex, instead of an exclusion complex, as **CB6** and **CB7** bear the same functional groups on the outer surface. This conclusion is also supported by the DFT studies, which show the encapsulation of neutral **APO** and **DiS-NH$_2$** in **CB7**. Control experiments with ten equivalents of acetone and ten equivalents of methanol were carried out to confirm that the residues in cucurbiturils do not affect the kinetic results (Figure S8). In case of **CB7**, experimental acetone content present 1:0.06 ratio, while **CB6** present a 1:1.09 acetone ratio. Control experiment were carried out using a ten times higher concentration than present in the sample. Thus, **DiS-NH$_2$** showed the same behavior as **APO**, with a 1:1 **CB7** ratio contributing to stability and the addition of **CB6** promoting degradation to 2-aminobenzenethiolate.

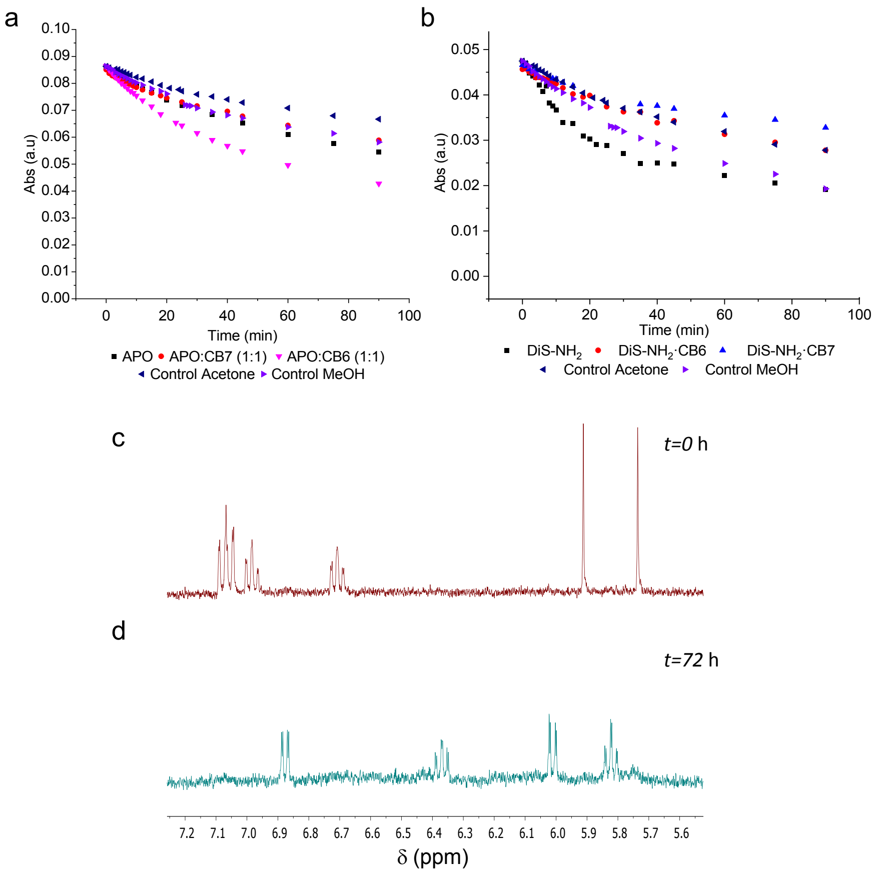

Figure S9. Kinetic study of (a) **APO** (5 μM) and (b) **DiS-NH$_2$** (40 μM) in basic media (pH 10.0). ^{1}H NMR spectra of **APO** (1 mM) at (c) initial time and (d) after 72 h, at pD 14.2. Both experiments have been developed in pure H_2O (mili-Q quality), at 25ºC, and the basic media have been adjusted by adding NaOH.

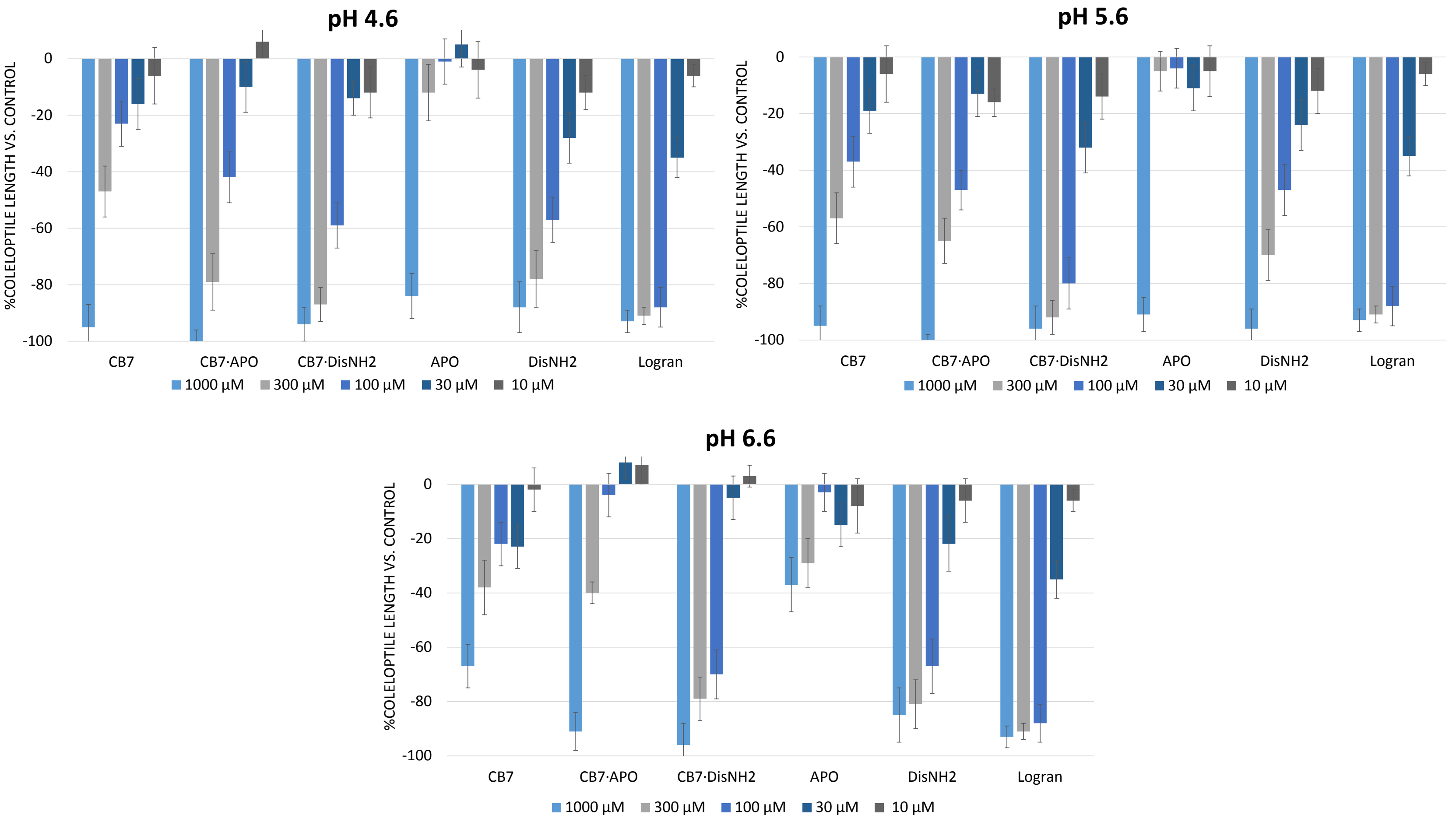

Figure S10. Results of *in vitro* bioassay over wheat coleoptile at different pH media. **CB7**, **APO, DiS-NH$_2$**, their complexes and positive control Logran® were tested.

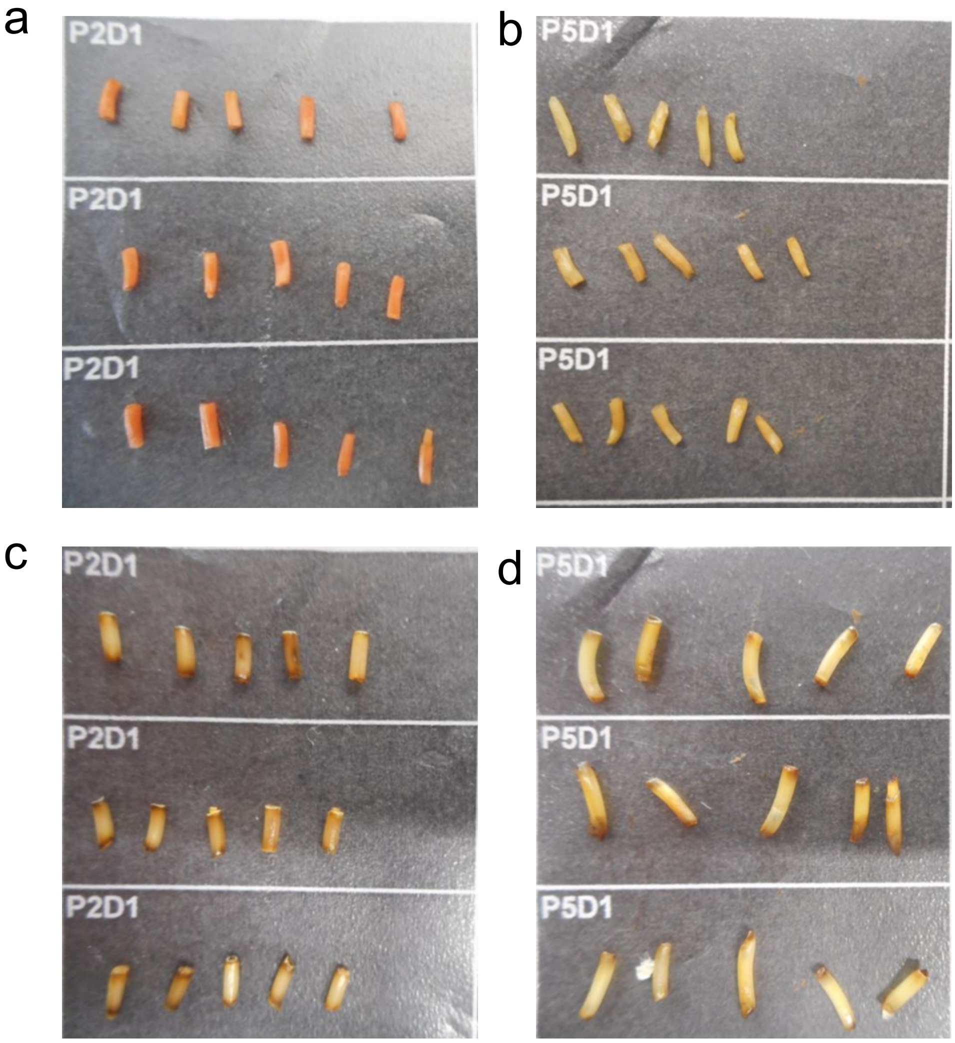

Figure S11. Images of wheat coleoptile after 24 h of treatment at (a and b) pH 4.6 and (c and d) pH 5.6, with (a and c) **CB7·APO** and (b and d) **APO**.
